# Beyond the market: Food safety knowledge and practices among female household food handlers in the domestic kitchen

**DOI:** 10.1371/journal.pone.0356599

**Published:** 2026-09-02

**Authors:** Edward Ken Essuman, Afia Sakyiwaa Amponsah, Vida Gyimah Boadu, Evangeline Adu Okraku

**Affiliations:** 1 School of Allied Health Sciences, Department of Nutrition and Dietetics, University of Health and Allied Sciences, Ho, Ghana; 2 Department of Hospitality and Tourism, Sunyani Technical University, Sunyani, Ghana; 3 Department of Hospitality and Tourism Education, University of Skills Training and Entrepreneurial Development, Kumasi, Ghana; Lusofona University of Humanities and Technologies: Universidade Lusofona de Humanidades e Tecnologias, PORTUGAL

## Abstract

Food safety within the household remains a critical yet often overlooked component of public health, particularly in developing countries where food preparation is largely managed at the domestic level. Despite high levels of formal education in urban populations, knowledge does not consistently translate into safe food-handling behaviour. This study assessed food safety knowledge, perceptions, and practices among female household food handlers in the Ho Municipality of the Volta Region, Ghana. A community-based cross-sectional design was employed, involving 386 female household food handlers aged 18 years and above, selected through a combined purposive and systematic sampling procedure from households in Ho Municipality. Data were collected using a semi-structured questionnaire administered through face-to-face interviews. Knowledge was assessed across five items; participants who answered three or more correctly were classified as having good knowledge. Descriptive statistics, chi-square tests, and ordinal logistic regression were used to analyse data (SPSS version 22; p ≤ 0.05). The findings revealed generally high awareness of food safety: 81.9% reported practising personal hygiene during food preparation, and 86.5% recognised that poor hygiene contributes to food contamination. However, this awareness did not consistently translate into safe practices. Only 22.5% reported that they regularly wash foodstuffs before usage, and 42.2% wash their hands before cooking. Risky behaviours were common, including tasting food with bare hands (43.8%) and using the same ladle throughout cooking (31.1%). Additionally, 60.1% were unaware of the correct freezer temperature (−18°C). Educational level was the only factor significantly associated with food safety knowledge (p = 0.001) and remained the sole independent predictor after logistic regression adjustment (adjusted OR 3.66, 95% CI 1.72–7.79, p = 0.001 for tertiary versus primary-educated respondents). A clear gap exists between food safety knowledge and practice among female household food handlers in Ho Municipality. Targeted, practical, and culturally appropriate interventions that promote behaviour change, rather than awareness alone, are needed to reduce preventable food-borne illness, particularly among children.

## Introduction

Food is a fundamental human need, essential for growth, health, and survival. In most households, particularly in developing countries, food preparation and handling are primarily undertaken by women, who often simultaneously manage multiple responsibilities, including childcare and maintaining the home environment. As individuals who are often directly responsible for food preparation and serving within the household, their knowledge, perceptions, and practices play a critical role in ensuring food safety and protecting household health, especially among children. Unlike commercial settings where food safety is regulated and monitored, the domestic kitchen relies largely on individual awareness and behaviour, making household food-handling practices important for preventing food-borne illness.

Food safety extends beyond basic food hygiene. While food hygiene emphasises cleanliness, food safety encompasses the broader conditions and measures required during food production, storage, preparation, and serving to ensure that food is safe for consumption [1]. In domestic kitchens, which are often multipurpose spaces, the risk of contamination can be heightened by poor personal hygiene, improper storage, inadequate cooking, and cross-contamination between raw and cooked foods. Studies have shown that contamination frequently occurs when food is insufficiently heated, stored at inappropriate temperatures, handled by infected individuals, or prepared on contaminated surfaces [2].

Food-borne illness remains a major global public health concern. The World Health Organization (WHO) estimates that approximately 600 million people fall ill annually following consumption of contaminated food [3]. Reports indicate that 30–40% of food-borne disease cases occur in the home [4]. In developing countries, approximately 2.2 million deaths annually are attributed to food-borne illnesses, with an estimated 1.9 million of these deaths occurring among children [5]. Children are particularly vulnerable due to their immature immune systems and lower body weight, making safe food handling at home a critical preventive strategy. In sub-Saharan Africa, including Ghana, diarrhoeal disease, often linked directly or indirectly to contaminated food and water, ranks among the leading causes of outpatient visits [1]. Globally, contaminated food contributes to billions of diarrhoeal cases and millions of deaths annually [6].

Although substantial research has examined food safety practices among food vendors and commercial food establishments, highlighting issues such as poor hygiene, inadequate protective measures, and unsafe handling practices [7–9], relatively little attention has been paid to women and caregivers in domestic settings. This distinction is important. Food vendors operate within regulatory frameworks and public scrutiny, whereas household food preparation is private, less monitored, and deeply influenced by cultural practices, personal beliefs, and knowledge levels. Interventions designed for commercial food handlers may therefore not adequately address the unique dynamics of home kitchens.

Understanding food safety knowledge and practices among household food handlers is essential for designing effective interventions aimed at reducing food-borne illnesses, particularly among children. In Ghana, as in many parts of sub-Saharan Africa, women often bear primary responsibility for household food procurement, preparation, and storage, making them key contributors to domestic food safety [1]. Gender roles shape not only who cooks but also how food safety knowledge is acquired and applied; because female caregivers often play a central role in household food preparation and safety, they may represent an important target group for household-level food safety interventions [10]. Despite this, female household food handlers remain relatively understudied compared with commercial food handlers, and evidence from urban Ghanaian domestic settings is sparse. Previous studies in the Ghanaian context have focused predominantly on food vendors, school caterers, and institutional food handlers [7,11,12], and recent cross-sectional surveys across West Africa and the Eastern Mediterranean have confirmed that domestic food safety gaps persist even in relatively educated urban populations [13,14], leaving a gap in understanding how domestic food safety knowledge and behaviour are distributed among urban female caregivers in Ghana specifically. This gap is critical: home kitchens are unregulated, largely invisible to public health systems, and responsible for a substantial share of food-borne disease burden [4]. The present study addresses this gap by examining food safety knowledge, perceptions, and practices among female household food handlers in the Ho Municipality, Volta Region, Ghana. Specifically, it aimed to: (1) describe the socio-demographic profile of female household food handlers; (2) assess their food safety knowledge, perceptions, and practices; and (3) identify socio-demographic predictors of good food safety knowledge. By focusing on the domestic sphere rather than commercial outlets, this study highlights the home as an important yet underexplored setting for food safety interventions, and emphasises the need for targeted education and awareness programmes tailored to caregivers in resource-constrained settings.

## Materials and methods

### Study design

This study employed a community-based cross-sectional design to assess food safety knowledge, perceptions, and practices among female household food handlers in the Ho Municipality of the Volta Region, Ghana. A cross-sectional approach was considered appropriate because it permitted the collection of data from a defined population at a single point in time, providing a snapshot of prevailing food safety practices within households.

### Study area

The study was conducted in Ho Municipality, one of the administrative districts in the Volta Region of Ghana. Ho serves as the regional capital and is geographically situated between Mount Adaklu and Mount Galenukui. The municipality shares boundaries with Adaklu and Agotime-Ziope to the south, Ho West District to the north and west, and the Republic of Togo to the east. According to the 2021 Population and Housing Census, Ho Municipality has a population of approximately 198,000, with females constituting 51.2% [15].

Ho Municipality has 29 health facilities, including a regional hospital, a district hospital, a polyclinic, health centres, Community-based Health Planning and Services (CHPS) compounds, private hospitals, and clinics. The predominant occupation in the municipality is agriculture. Households commonly cultivate crops such as yam, cassava, and green leafy vegetables, and rear animals including goats, sheep, and poultry. Two major market centres operate within the municipality, improving access to fresh food produce. These contextual characteristics make Ho Municipality an appropriate setting for examining domestic food safety practices, particularly in homes where food is often sourced locally and prepared daily.

### Study population

The target population comprised female household food handlers aged 18 years and above residing in Ho Municipality who prepare meals at home.

#### Inclusion and exclusion criteria.

Women aged 18 years and above who were actively involved in cooking or food preparation within their households and who provided written informed consent were eligible to participate. Women below 18 years were excluded from the study due to their legal minor status and to ensure participants could provide fully informed consent independently.

#### Sample size determination.

The sample size was calculated using the Raosoft sample size calculator [16]. Assuming a 95% confidence level, a 5% margin of error, a response distribution of 50%, and the 2021 PHC population of approximately 198,000 for Ho Municipality, a minimum sample size of 383 participants was estimated. A total of 386 participants were recruited.

#### Sampling procedure.

A combined purposive-systematic sampling procedure was employed. The study purposively targeted female household food handlers as the group most responsible for domestic meal preparation in the Ghanaian context; this eligibility criterion constituted the purposive component of sampling. Ho Municipality was then divided into its constituent electoral areas, from which eight areas were selected to achieve geographic spread across peri-urban and urban zones. Within each selected area, data collectors used systematic interval sampling: beginning at a randomly identified household within each area, every third household thereafter was approached. In each household, the adult female most responsible for daily meal preparation was invited to participate. Where more than one eligible woman resided in a household, the primary cook was selected. Households were revisited once if no eligible resident was present on the first visit. This combined approach ensured that participants were genuinely involved in domestic food preparation while also achieving systematic geographic coverage across the municipality.

#### Data collection procedure.

Data were collected using a semi-structured questionnaire administered through face-to-face interviews from August 8, 2021 to October 27, 2021. The principal investigator and a trained research assistant visited selected households between 8:00 a.m. and 2:00 p.m. each day. The purpose and nature of the study were explained to eligible participants, and written informed consent was obtained prior to participation. English was the primary language of communication; where necessary, local language interpretation (Ewe) was provided to ensure clarity and accurate responses. Each interview lasted approximately 15 minutes.

#### Instrumentation.

The questionnaire was structured into five sections:

- Section A: Socio-demographic characteristics (age, educational level, occupation, number of children, marital status, monthly income).
- Section B: Perceptions and practices related to personal hygiene and food safety (14 items addressing hygiene, handwashing, food handling, and sanitation).
- Section C: Knowledge of food safety at home (5 questions with response options: “Yes,” “No,” and “I don’t know,” to minimize guessing).
- Section D: Behaviour during food preparation and storage (e.g., tasting food while cooking, covering hair, handling leftovers, waste disposal).
- Section E: Perceptions and practices regarding kitchen management and food storage (e.g., washing vegetables, cleaning utensils, storing frozen foods, preserving meat, handling defrosted foods).

The instrument was designed to capture both knowledge and practical behaviours related to domestic food safety. It was developed de novo by the research team, drawing on item structures reported in validated instruments used in comparable sub-Saharan African settings [11,17]. A panel of three food scientists and a public health nutritionist reviewed all items for content validity, and the instrument was revised iteratively to ensure alignment with the study objectives and the local cultural context. The questionnaire was pre-tested on 20 non-participants in a neighbouring community and revised for clarity before field deployment. Internal consistency was assessed using Cronbach’s alpha (SPSS Reliability Analysis procedure). The overall instrument yielded a Cronbach’s alpha of 0.71, indicating acceptable internal consistency. The knowledge subscale (Section C, five items) returned an alpha of 0.68, and the practice subscale (Sections B, D, and E) returned an alpha of 0.74, both within the acceptable range (alpha ≥0.60) for exploratory social and health research [18].

### Knowledge scoring

Knowledge of food safety was assessed using the five items in Section C. Each correctly answered item was scored 1 point. Participants who correctly answered three or more items (score ≥3 out of 5, i.e., ≥ 60%) were classified as having “good knowledge” for descriptive and bivariate purposes; those who answered fewer than three correctly were classified as having “poor knowledge.” This threshold is consistent with similar studies in sub-Saharan Africa [11,17]. For multivariate analysis, the raw knowledge score (0–5) was retained as an ordered outcome variable to avoid information loss from dichotomisation, and ordinal logistic regression was applied accordingly.

### Income classification

Monthly income was categorised in line with approximate Ghanaian national wage benchmarks at the time of data collection: low income (<GHS 500/month), lower-middle income (GHS 500–1,499/month), upper-middle income (GHS 1,500–2,999/month), and high income (≥ GHS 3,000/month).

### Ethical considerations

Ethical clearance for this study was obtained from the University of Health and Allied Sciences Research Ethics Committee (Approval No.: UHAS-REC-A.7 [16] 21–22). Written informed consent was obtained from all participants prior to data collection. Participation was voluntary, and respondents were assured of the confidentiality of their responses. No personally identifiable information was collected or retained beyond the study period.

### Data Analysis

Data were entered and analysed using Microsoft Excel (version 14.0) and SPSS (version 22). Descriptive statistics were generated and findings presented in tables to summarise the distribution of responses. The chi-square (χ²) test was used to examine bivariate associations between socio-demographic characteristics and food safety knowledge. Ordinal logistic regression was subsequently performed to identify independent predictors of food safety knowledge score, with results reported as adjusted odds ratios (AOR) with 95% confidence intervals (CI). Ordinal logistic regression was selected in preference to binary logistic regression because the knowledge outcome (score range 0–5) is an ordered polytomous variable rather than a strictly dichotomous one; this approach retains the full information in the score distribution and avoids the information loss inherent in arbitrary dichotomisation [19]. The proportional odds assumption was verified using the Brant test prior to model fitting. All variables with p < 0.20 in the chi-square analysis were included in the regression model. A p-value of ≤ 0.05 was considered statistically significant throughout.

## Results and discussion

### Socio-demographic characteristics of respondents

A total of 386 female household food handlers participated in the study. The majority (38.3%) were between 21 and 26 years of age, followed by those aged 27–31 years (23.6%) and 18–20 years (23.1%), as shown in Table 1. Only 3.9% were above 45 years, indicating that most participants were young adults actively involved in childcare and household food preparation.

**Table 1:** Socio-demographic characteristics of respondents (N = 386).

Demography		Frequency	Percent (%)
Age	18–20	89	23.1
	21–26	148	38.3
	27–31	91	23.6
	32–45	43	11.0
	>45	15	3.9
Educational Level	No formal education	25	6.5
	Primary	51	13.2
	Secondary	132	34.2
	Tertiary	178	46.1
Occupation	Teacher	76	19.7
	Farmer	37	9.6
	Nurse	32	8.3
	Others	241	62.4
Number of Children	None	89	23.1
	One child	118	30.6
	Two children	97	25.1
	Three or more	82	21.2

Educationally, nearly half (46.1%) had attained tertiary education, while 34.2% had secondary education. Only 6.5% had received no formal education. This relatively high level of educational attainment is noteworthy. However, food-safety education remains important for improving awareness among household food preparers [10]. However, as the findings below demonstrate, formal education alone does not guarantee safe behaviour in the domestic kitchen.

Regarding occupation, 62.4% were engaged in activities categorised as “other” employment, alongside teachers (19.7%), farmers (9.6%), and nurses (8.3%). In terms of family size, 30.6% had one child and 25.1% had two children. These characteristics indicate that many participants balance employment with caregiving and meal preparation, both of which may compete for time and attention in ways that affect food safety practice.

### Perception and practice of personal hygiene

The findings indicate varying levels of adherence to recommended food hygiene practices (Table 2). Most respondents (81.9%) reported awareness of personal hygiene practices during food preparation, and 86.5% agreed that poor personal hygiene can lead to food contamination, reflecting a generally positive attitude toward hygiene as a preventive measure against food-borne illness. Similar patterns have been observed among food vendors in Accra, where awareness of hygiene principles was high but actual compliance was inconsistent [12].

**Table 2:** Food hygiene and safety practices among respondents (N = 386).

Variable	Response n (%)
Practices personal hygiene during food preparation	Yes: 316 (81.9); No: 70 (18.1)
Washes foodstuffs before preparation	Yes: 87 (22.5); No: 299 (77.5)
Reheats cold foods to safe temperature before eating	Yes: 27 (7.0); No: 359 (93.0)
Washes hands before cooking	Yes: 163 (42.2); No: 223 (57.8)
Covers hair when cooking	Yes: 59 (15.3); No: 327 (84.7)
Avoids going barefoot in the kitchen	Yes: 13 (3.4); No: 373 (96.6)
Cleans the kitchen environment regularly	Yes: 106 (27.5); No: 280 (72.5)
Covers foodstuffs during preparation	Yes: 106 (27.5); No: 280 (72.5)
Cooks food in a clean environment	Yes: 18 (4.7); No: 368 (95.3)
Cleans surface areas before use	Yes: 31 (8.0); No: 355 (92.0)
Agrees poor personal hygiene contributes to food contamination	Yes: 334 (86.5); No: 52 (13.5)
Washes hands with soap and water	Yes: 287 (74.4); No: 21 (5.4); Sometimes: 78 (20.2)
Frequency of handwashing during food handling	Three times: 92 (23.8); Six times: 45 (11.7); After every procedure: 249 (64.5)
Cleaning kitchen surfaces can reduce risk of illness	Agree: 304 (78.8); Not sure: 46 (11.9); Don’t know: 36 (9.3)

Despite this general awareness, several specific hygiene practices were not consistently followed. Only 22.5% of respondents reported regularly washing foodstuffs before preparation, and only 7.0% reported reheating cold food to a safe internal temperature before consumption, a practice critical for destroying pathogens that may have proliferated during storage. Strikingly, while 81.9% reported practising personal hygiene during food preparation, only 4.7% reported cooking food in a clean environment and 8.0% reported cleaning surfaces before use. This apparent contradiction likely reflects a disconnect between respondents’ general self-concept as hygienic individuals and their awareness of specific surface-decontamination requirements. From a Health Belief Model perspective, respondents may perceive personal hygiene as a broad, positive trait (perceived benefit) without perceiving kitchen surface contamination as a personally relevant threat (low perceived susceptibility), thereby failing to translate general hygiene identity into targeted surface-cleaning behaviour [20]. This pattern is consistent with findings from Lebanon and other Eastern Mediterranean contexts, where high general hygiene awareness coexisted with low compliance on specific tasks such as surface sanitisation and produce washing [13]. Addressing this gap requires interventions that shift perceived susceptibility as well as knowledge, for instance, by making the microbial hazards on kitchen surfaces tangible through community demonstration activities. Hand hygiene was more widely reported: 74.4% stated they washed hands with soap and water, and 64.5% washed hands after every food-handling procedure. Handwashing is consistently identified as one of the most effective individual measures to reduce microbial transmission [21]; however, global evidence shows that compliance with correct handwashing technique remains inconsistent even among populations with good knowledge [22]. In the present study, only 42.2% specifically reported washing hands before cooking, and just 15.3% covered their hair during food preparation. These gaps illustrate the well-documented disconnect between food safety knowledge and practice [23] and reflect the critical role of perceived behavioural control in the Theory of Planned Behaviour: even where intention and knowledge are present, competing time pressures and habitual routines in the domestic kitchen can suppress safe behaviour [24].

Regarding the frequency of handwashing during food preparation, most respondents (64.5%) washed their hands after every procedure, while 23.8% did so three times and 11.7% six times during food preparation. Furthermore, 78.8% agreed that maintaining a clean kitchen surface can reduce the risk of illness, reflecting an understanding that the kitchen environment itself is a critical node of food safety.

### Knowledge of food safety

The majority (88.6%) correctly defined food hygiene as actions taken to ensure food is properly handled, stored, prepared, and served safely (Table 3). This high awareness of the conceptual definition is encouraging, though it must be interpreted alongside the practical gaps discussed elsewhere. Furthermore, 69.2% acknowledged that skin infections can contaminate food, consistent with evidence that open wounds and skin lesions may introduce pathogens such as Staphylococcus aureus into meals [25–27].

**Table 3:** Knowledge of food hygiene and food safety among respondents (N = 386).

Variable	Response	n (%)
Understanding of food hygiene	Actions taken to ensure food is properly handled, stored, prepared, and served.	342 (88.6)
	Maintaining cleanliness of the immediate environment.	32 (8.3)
	General environmental cleanliness only.	12 (3.1)
Can skin infections contaminate food?	Yes	267 (69.2)
	No	119 (30.8)
Knowledge that consuming food from swollen cans may be harmful	Yes	286 (74.1)
	No	100 (25.9)
Importance of discarding expired foods	Yes	345 (89.4)
	No	17 (4.4)
	Don’t know	24 (6.2)
Feeding children food warmed to a safe temperature is beneficial	Yes	264 (68.4)
	No	32 (8.3)
	Sometimes	90 (23.3)

Knowledge about food spoilage was generally strong. Most participants (89.4%) recognised the importance of discarding expired foods, and 74.1% correctly identified that consuming food from swollen cans can be harmful, demonstrating reasonable awareness of visible spoilage indicators. Previous research has reported an association between educational attainment and knowledge of food labelling and expiry dates [28]. The near-universal recognition of the need to discard expired food in this highly educated sample is therefore consistent with this relationship.

Feeding children food warmed to a safe temperature was considered good practice by 68.4% of respondents. While keeping food warm can inhibit bacterial multiplication when temperatures exceed 60°C, it is important to distinguish safe warming (maintaining food above the danger zone, 5–60°C) from perfunctory reheating, which may not achieve sufficient internal temperatures to eliminate pathogens [29]. Future instruments should probe the specific temperatures respondents target during reheating, rather than the practice in general. The public health consequences of these knowledge gaps are substantial. Food-borne pathogens such as Salmonella, Staphylococcus aureus, and Listeria monocytogenes proliferate rapidly between 5°C and 60°C, and inadequate temperature management in the domestic kitchen is a recognised driver of household food-borne illness [29,30]. In Ghana, where diarrhoeal disease accounts for a significant proportion of childhood morbidity [1], correcting temperature management knowledge through community-level food safety education represents a low-cost, high-impact intervention opportunity.

### Knowledge and behaviour related to food preparation and storage

Results on food preparation and storage practices revealed notable risk behaviours alongside some positive habits (Table 4). Nearly half of the respondents (47.7%) washed eggs with water before cracking them. While washing visibly soiled eggs can reduce surface contamination, wet washing may, if performed incorrectly, facilitate bacterial entry through the porous shell; dry-wiping or purchasing pre-washed eggs is generally preferred in commercial settings [31]. In addition, 43.8% reported tasting food by placing it on the palm, and 31.1% used the same ladle throughout cooking; both practices that create direct oral-to-food transmission pathways and increase the risk of cross-contamination [23]. Hair covering during cooking was practised by only 40.4% of respondents, indicating limited adoption of physical barriers to food contamination.

**Table 4:** Respondents’ knowledge and practices related to food preparation and storage (N = 386).

Variable	Response	Frequency	Percent (%)
Handling eggs before use	Clean with napkin	74	19.2
	Wash with water	184	47.7
	Wash with soap and water	36	9.3
	Crack without washing	92	23.8
Method of tasting food during cooking	Taste with the same ladle used for cooking	120	31.1
	Taste with a separate tablespoon	97	25.1
	Place food on palm and taste	169	43.8
Covering hair during cooking	Yes	156	40.4
	No	230	59.6
Storage of leftover food	Cover and keep at a safe place	111	28.8
	Leave uncovered	36	9.3
	Cover and store in refrigerator	239	61.9
Frequency of disposing kitchen waste	Once a day	87	22.5
	After every cooking activity	116	30.1
	After daybreak	94	24.4
	When the bin is full	89	23.1
Handling uneaten leftover food	Discard immediately	107	27.7
	Refrigerate and reheat to safe temperature before consumption	196	50.8
	Keep in the kitchen and reheat before consumption	74	19.2
	Eat if it smells acceptable	9	2.3
Cleaning kitchen countertop/stove	Clean with a dry rag	63	16.3
	Clean with a wet rag	142	36.8
	Clean with detergent and warm water	74	19.2
	Use all the above methods	107	27.7
Correct way of washing dishes	Soak for hours and wash with the same water	16	4.1
	Wash immediately after a meal	256	66.3
	Wash in basin and dry with dishcloth	114	29.5
Optimal temperature for frozen food storage	4°C	39	10.1
	0°C	94	24.4
	−18°C (correct)	21	5.4
	Do not know	232	60.1
Preservation of raw meat	Store directly in freezer	139	36.0
	Smoke it	61	15.8
	Steam it	51	13.2
	Slice, seal, and store in freezer	135	35.0
Refreezing of defrosted meat	Yes	201	52.1
	No	39	10.1
	Maybe	75	19.4
	Do not know	71	18.4

With regard to food storage, encouragingly, 61.9% reported covering leftover food and storing it in the refrigerator, and 50.8% indicated they refrigerate leftovers immediately and reheat them before consumption. Proper refrigeration is critical, as domestic refrigerators frequently operate above recommended temperatures, potentially allowing bacterial proliferation even in stored food [32].

Waste management practices were mixed. Approximately 30.1% disposed of kitchen waste after every cooking activity, while 23.1% waited until the bin was full. Infrequent waste disposal is associated with pest infestation and environmental contamination, both of which increase food safety risk. The most common kitchen surface cleaning method was a wet rag (36.8%), and only 19.2% used detergent and warm water, a combination more effective at removing grease residues and reducing microbial load. Dish washing was more consistent: 66.3% reported washing dishes immediately after meals.

A notable knowledge gap concerned frozen food storage temperature: 60.1% of respondents did not know that −18°C is the recommended temperature for frozen food storage. Temperature control is a most fundamental principle of food preservation, as temperatures below −18°C inhibit the growth of virtually all food spoilage microorganisms [30,31]. This gap is significant given that 88.6% of the same respondents could correctly define food hygiene, reinforcing the knowledge–practice disconnect central to this study.

Regarding raw meat preservation, 36.0% stored meat directly in the freezer and 35.0% opted for slicing and sealing before freezing; both approaches are acceptable when implemented correctly. However, 52.1% believed that defrosted meat could be refrozen. Safe refreezing requires that the thawed food has remained at refrigerator temperature throughout (i.e., ≤ 4 °C) and has not entered the danger zone (5–60°C); if thawing has occurred at room temperature, refreezing presents microbiological risks even if the food appears unspoiled [33,34]. This nuance was likely not appreciated by the majority of respondents who agreed to refreezing.

Cross-contamination practices also represent a significant hazard: only 36.8% correctly identified that the same cutting board should not be used simultaneously for raw and cooked foods. Misuse of cutting boards is one of the most commonly reported domestic food safety errors globally [35], with similarly low compliance (30–40%) documented among household food handlers in South Africa and Haiti [36]. Vegetable washing practices varied: 39.1% used clean water and 39.1% used salt and water, while very few used hot water or vinegar, approaches sometimes advocated in low-resource settings to reduce microbial load on fresh produce [37]. These findings are broadly consistent with the South African study by Masai et al. [17], which found that surface decontamination and cold-chain management were the weakest domains of domestic food safety knowledge across income levels. Reinforcing that these are not context-specific shortcomings but a structural feature of informal household food safety education globally.

### Association between socio-demographic characteristics and food safety knowledge

The associations between socio-demographic characteristics and food safety knowledge are presented in Table 5. Educational level was the only variable significantly associated with food safety knowledge at p ≤ 0.05 (p = 0.001). This finding is consistent with [11] who reported a significant association between educational level and food safety knowledge among institutional food handlers in Ghana (p < 0.05), and with [38] who found that schooling and training exposure significantly predicted food safety knowledge and attitudes among food handlers in Brazil. Higher levels of formal education may be associated with greater literacy and critical-thinking skills, which could facilitate interpretation and application food-safety information.

**Table 5:** Descriptive association between socio-demographic characteristics and food safety knowledge (good vs poor) among household food handlers (N = 386).

Variable	Category	Good Knowledge n (%)	Poor Knowledge n (%)	p-value
Age Group (years)	18–25	51 (57.3)	38 (42.7)	0.220
	26–35	83 (56.1)	65 (43.9)	
	36–45	42 (46.2)	49 (53.8)	
	≥46	26 (44.8)	32 (55.2)	
Educational Level	No formal education	15 (60.0)	10 (40.0)	0.001*
	Primary education	14 (27.5)	37 (72.5)	
	Secondary education	70 (53.0)	62 (47.0)	
	Tertiary education	103 (57.9)	75 (42.1)	
Marital Status	Single	44 (57.9)	32 (42.1)	0.083
	Married	13 (35.1)	24 (64.9)	
	Cohabiting	20 (62.5)	12 (37.5)	
	Divorced/Widowed	125 (51.9)	116 (48.1)	
Monthly Income Level†	Low income (<GHS 500)	57 (64.0)	32 (36.0)	0.056
	Lower-middle (GHS 500–1,499)	61 (51.7)	57 (48.3)	
	Upper-middle (GHS 1,500–2,999)	48 (49.5)	49 (50.5)	
	High income (≥GHS 3,000)	36 (43.9)	46 (56.1)	

* Statistically significant at p ≤ 0.05. † Income thresholds based on prevailing Ghanaian national wage benchmarks at time of data collection.

Of note, the relationship between educational level and good knowledge was not monotonically increasing: respondents with no formal education showed a proportionally higher rate of good knowledge (60.0%) than those with primary education only (27.5%), followed by a recovery at secondary (53.0%) and tertiary levels (57.9%). This non-linear pattern may reflect the small sample size in the no-formal-education group (n = 25), which limits the stability of this estimate, or it may reflect practical knowledge acquired through oral tradition and lived experience in the absence of formal schooling, a finding that warrants further qualitative investigation.

Age, marital status, and monthly income were not statistically significant in the bivariate analysis. However, marital status (p = 0.083) and monthly income (p = 0.056) showed near-significant trends, suggesting they may exert some influence on food safety knowledge and merit inclusion in multivariate analyses.

### Ordinal logistic regression: Independent predictors of food safety knowledge

**Fig 1:**
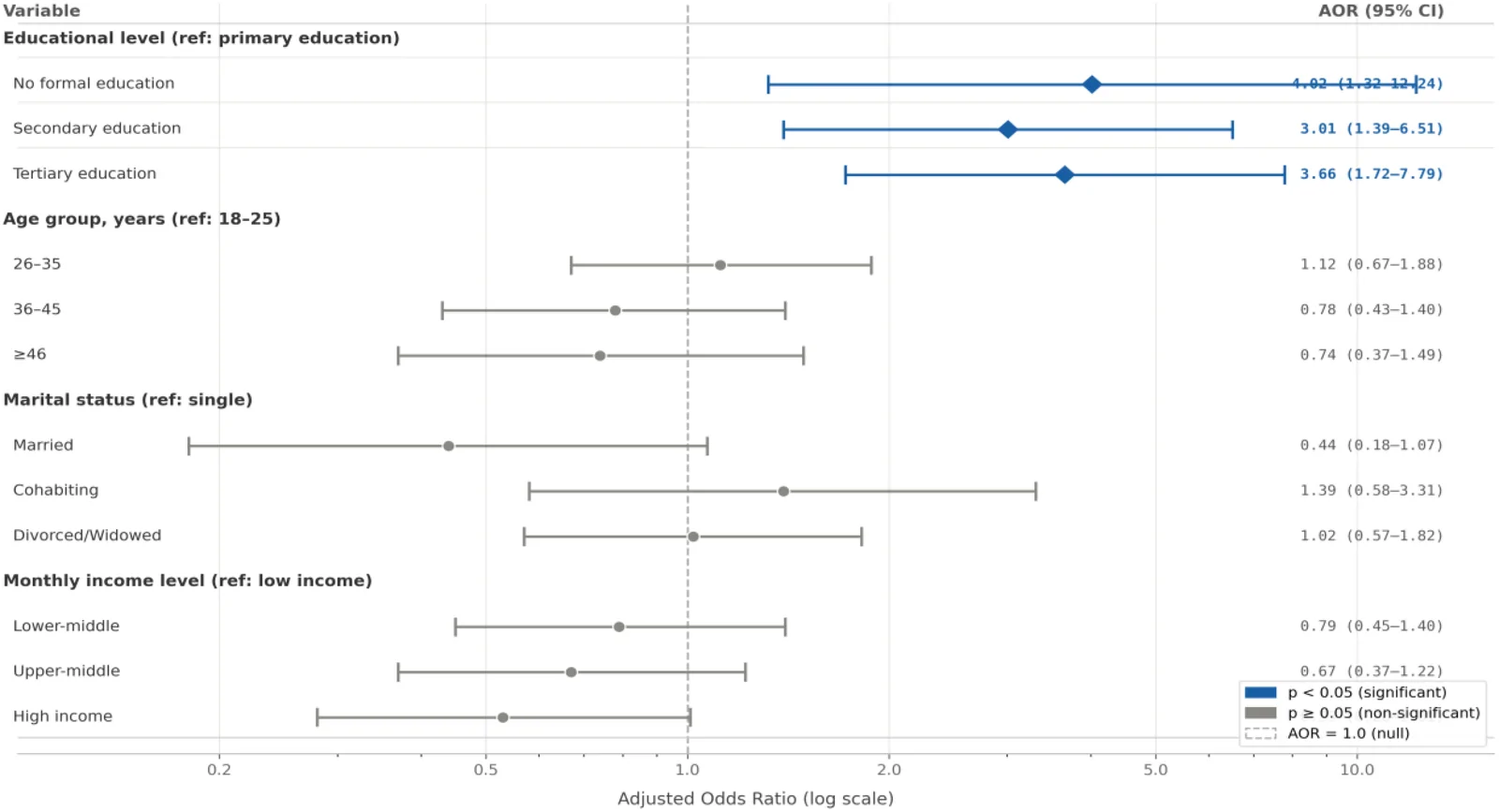
Binary Logistic Regression Predictors of Good Food Safety Knowledge.

To identify independent predictors of food safety knowledge score while controlling for potential confounders, ordinal logistic regression was performed using the raw knowledge score (0–5) as the ordered outcome variable. All variables with p < 0.20 in the chi-square analysis (educational level, marital status, age group, and monthly income) were entered into the model. Results are presented in Table 6 and Fig 1. Educational level remained the sole statistically significant independent predictor of food safety knowledge score after adjustment. Compared to respondents with primary education only, those with secondary education (AOR 3.01, 95% CI 1.39–6.51, p = 0.005), tertiary education (AOR 3.66, 95% CI 1.72–7.79, p = 0.001), and no formal education (AOR 4.02, 95% CI 1.32–12.24, p = 0.014) all had significantly higher odds of good knowledge. The consistency of this effect across both secondary and tertiary levels indicates that even secondary schooling provides a meaningful threshold of food safety understanding, with direct implications for basic education policy as a public health instrument. Respondents with tertiary education had the highest adjusted odds of good knowledge (AOR 3.66), aligning with an extensive literature linking higher formal education to stronger health literacy, greater engagement with food safety information, and improved capacity to translate knowledge into practice [10,11,38].

**Table 6:** Ordinal logistic regression of predictors of food safety knowledge score among household food handlers (N = 386).

Variable	Category	AOR	95% CI	p-value
Age Group (years)	18–25 (Ref)	1.00	—	
	26–35	1.12	0.67–1.88	0.659
	36–45	0.78	0.43–1.40	0.401
	≥46	0.74	0.37–1.49	0.400
Educational Level	Primary (Ref)	1.00	—	
	No formal education	4.02	1.32–12.24	0.014*
	Secondary	3.01	1.39–6.51	0.005**
	Tertiary	3.66	1.72–7.79	0.001**
Marital Status	Single (Ref)	1.00	—	
	Married	0.44	0.18–1.07	0.070
	Cohabiting	1.39	0.58–3.31	0.460
	Divorced/Widowed	1.02	0.57–1.82	0.944
Monthly Income Level	Low income (Ref)	1.00	—	
	Lower-middle	0.79	0.45–1.40	0.422
	Upper-middle	0.67	0.37–1.22	0.190
	High income	0.53	0.28–1.01	0.053

AOR: Adjusted Odds Ratio from ordinal logistic regression (PLUM procedure, SPSS v.22); CI: Confidence Interval; Ref: Reference category. Proportional odds assumption verified (Brant test p > 0.05 for all predictors). * p ≤ 0.05; ** p ≤ 0.01.

The elevated odds ratio for the no-formal-education group (AOR 4.02, 95% CI 1.32–12.24) is a notable and somewhat unexpected finding that warrants careful interpretation. The wide confidence interval reflects the small cell size (n = 25) and limits the precision of this estimate. Nevertheless, similar patterns have been observed in sub-Saharan African settings, where practical food-handling knowledge is effectively transmitted through oral tradition, community networks, and extended household practice in the absence of formal schooling [17,37]. It is possible that women without formal education in this sample had accumulated grounded, experience-based food safety knowledge through daily domestic practice and intergenerational transfer, while those who reached only primary level received insufficient formal health information and no longer had full access to traditional knowledge systems. This hypothesis warrants further qualitative exploration. Age, marital status, and monthly income were not statistically significant in the adjusted model. A near-significant inverse trend for high versus low income (AOR 0.53, 95% CI 0.28–1.01, p = 0.053) may reflect greater reliance on domestic workers in wealthier households, reducing the primary food handler’s direct engagement with food preparation and the knowledge-building that accompanies it. This pattern is consistent with observations in other low- and middle-income country contexts [17] and merits investigation in future studies with larger, income-stratified samples.

Fig 1 visually confirms the regression results: educational level is the only statistically significant independent predictor of food safety knowledge after adjustment for age, marital status, and monthly income. All three education categories above the reference level (primary education) returned odds ratios substantially greater than 1.0, with confidence intervals that do not cross the null. The non-significance of age (all p > 0.20) indicates that food safety knowledge may not be explained by domestic experience alone and may also be influenced by formal education and exposure to targeted health. The near-significant inverse income trend (high income AOR 0.53, 95% CI 0.28–1.01, p = 0.053) likely reflects greater delegation of food preparation to domestic workers in wealthier households, consistent with observations elsewhere in the region [17]. These findings have clear policy implications. Integrating food safety content into school health curricula at both primary and secondary levels offers the broadest population-wide reach. Community-based nutrition education programmes, delivered through CHPS compounds and women’s groups and tailored to women with limited formal schooling, are needed to complement school-based approaches and to reach those currently outside the formal education system.

### Limitations

Several limitations should be considered when interpreting these findings. First, the study employed a purposive sampling technique, which limits the statistical representativeness of the sample and restricts generalisation to the broader population of female household food handlers in Ho Municipality or the wider Volta Region. Future studies should adopt probability-based sampling frames to improve external validity.

Second, data were collected entirely by self-report. Social desirability bias may have led respondents to over-report safe food-handling practices, particularly where questions related to handwashing and kitchen hygiene, a concern heightened by the face-to-face interview format. The marked discrepancy between the high proportion who reported practising personal hygiene (81.9%) and the low proportions who reported cleaning surfaces (8.0%) or cooking in a clean environment (4.7%) may partly reflect this bias. Observational validation of self-reported practices through structured kitchen observations would provide a more objective assessment of actual behaviour and should be incorporated in future studies.

Third, the cross-sectional design precludes causal inference. The significant association between educational level and food safety knowledge cannot be interpreted as evidence of a causal pathway; reverse causality and confounding by unmeasured variables (e.g., exposure to food safety training, media use) cannot be excluded.

Fourth, the no-formal-education group comprised only 25 respondents (6.5%), which may have produced an unstable odds ratio estimate for this category in the logistic regression. This finding should be interpreted with caution and verified in larger samples with more balanced educational distributions.

Finally, this study assessed knowledge and self-reported practice but did not include microbiological sampling of food or surfaces in respondents’ kitchens. Future work combining survey methods with food safety audits or microbial testing would provide a more complete picture of the actual risk environment in domestic kitchens. Participant consent covered data sharing for research purposes, provided that no personally identifiable information is retained, which is the case for the dataset provided.

## Conclusion

This study demonstrates that female household food handlers in Ho Municipality possess generally good food safety knowledge and positive perceptions regarding hygiene, but that significant gaps exist between knowledge and actual kitchen practice. Risky behaviours, including improper food tasting methods, cross-contamination via shared utensils and cutting boards, and limited knowledge of correct freezing temperatures, were prevalent even among highly educated respondents. Educational level was the only independent predictor of good food safety knowledge in both bivariate and multivariate analyses, although near-significant trends for income suggest that socio-economic factors may also play a role.

These findings highlight that awareness campaigns alone are insufficient to change food-handling behaviour. Effective interventions must move beyond knowledge transmission to address the behavioural, cultural, and environmental determinants of unsafe practices in the domestic kitchen. Practical, skills-based food safety education, delivered through community health workers, women’s groups, and school curricula and tailored to the everyday realities of household food preparation in Ghana, may help to bridge the knowledge–practice gap and reducing preventable food-borne illness, particularly among children.

## Supporting Information

S1 Data Questionnaire. (DOCX)
