# Supplementary material for: Beyond the market: Food safety knowledge and practices among female household food handlers in the domestic kitchen
Source: PLoS One. 2026 Sep 2;21(9):e0356599. doi: 10.1371/journal.pone.0356599 (PMC13537588; doi:10.1371/journal.pone.0356599)
Supplement: Supplementary file 1 [file pone.0356599.s001.docx]

**QUESTIONNAIRE**

**FOOD SAFETY AND PERSONAL HYGIENE: PERCEPTIONS AND PRACTICES AMONG FEMALE HOUSEHOLD FOOD HANDLERS IN THE DOMESTIC KITCHEN**

**DATE OF INTERVIEW:** (DD/MM/YY)
**PLACE OF INTERVIEW:** __________________________
**INTERVIEWER ID:** ______________________________

**Section A: Demographic Data**

**1. Gender of respondent**

[ ] Male
[ ] Female

**2. Age of participant**

a. 18–20 years [ ]
b. 21–26 years [ ]
c. Above 26 years [ ]

**3. Educational level**

a. None [ ]
b. Primary [ ]
c. Secondary [ ]
d. Tertiary [ ]

**4. Occupation of participant**

a. Teacher [ ]
b. Farmer [ ]
c. Nurse [ ]
d. Other: __________________ [ ]

**5. Number of children**

a. One child [ ]
b. Two children [ ]
c. Three or more children [ ]

**Section B: Perceptions of Food Safety and Personal Hygiene**

**6. Are you aware of any personal hygiene practices to be followed during food preparation?**

a. Yes [ ]
b. No [ ]

**If the respondent answers “Yes,” proceed to Question 7.**

**7. Mention at least three good personal hygiene practices that you know.**

**Answer:** ___________________________________________________________

**8. Does poor personal hygiene contribute to food contamination?**

a. Yes [ ]
b. No [ ]

**9. What do you understand by food hygiene?**

a. Actions taken to ensure that food is handled, stored, prepared, and served in a manner that prevents contamination [ ]

b. Practices related to maintaining personal hygiene [ ]

c. Practices related to maintaining environmental cleanliness [ ]

**10. Can skin infections contaminate food?**

a. Yes [ ]
b. No [ ]

**11. Do you wash your hands with soap and water before handling food?**

a. Yes [ ]
b. No [ ]

**12. At what points do you wash your hands during food preparation?**
a. Before preparing food [ ]
b. After handling raw food [ ]
c. After using the toilet [ ]
d. After handling waste [ ]
e. Before serving food [ ]
f. Other: __________ [ ]

**13. Do you use hand sanitiser?**

a. Yes [ ]
b. No [ ]

**14. Keeping kitchen surfaces clean reduces the risk of illness. Do you agree?**

a. Agree [ ]
b. Not sure [ ]
c. Disagree [ ]

**Section C: Knowledge of Food Safety at Home**

**15. Are you aware that consuming food contaminated by dirty hands may cause illness?**

a. Yes [ ]
b. No [ ]

**16. Can the same cutting board be used for both raw and cooked foods?**

a. Yes [ ]
b. No [ ]
c. Don't know [ ]

**If No, why?**

**17. Do you think it is important to discard foods that have passed their expiry date?**

a. Yes [ ]
b. No [ ]
c. Don't know [ ]

**18. Do you feed your children warm food?**

a. Yes [ ]
b. No [ ]
c. Sometimes [ ]

**19. Before frying an egg, how do you handle the egg before cracking it?**

a. Clean it with a napkin [ ]
b. Wash it with water [ ]
c. Wash it with water and soap [ ]
d. Crack it without washing [ ]

**Section D: Food Preparation and Storage Practices**

**20. How do you taste food during cooking?**

a. Using the same ladle used to prepare the food [ ]
b. Using a tablespoon [ ]
c. Placing some of the food on the palm of the hand before tasting [ ]

**21. Do you cover food while cooking?**

a. Yes [ ]
b. No [ ]

**22. How do you store leftover food in the kitchen?**

a. Cover it and keep it in a safe place [ ]
b. Leave it uncovered [ ]
c. Cover it and store it in a refrigerator [ ]

**23. How often do you empty the kitchen bin?**

a. Once a day [ ]
b. After every cooking activity [ ]
c. After daybreak [ ]
d. When the bin is full [ ]

**Section E: Perceptions of Food Handling and the Use and Maintenance of Kitchen Facilities**

**24. How should vegetables be washed? Mention at least one method.**

**Answer:** ___________________________________________________________

**25. How do you handle leftover food that has not been completely eaten?**

a. Discard it immediately [ ]
b. Place it in the refrigerator immediately and reheat it before consuming [ ]
c. Store it in the kitchen and reheat it before consuming [ ]
d. Consume it as long as it smells good [ ]

**26. How do you clean your kitchen countertop and stove?**

a. With a dry cloth [ ]
b. With a wet cloth [ ]
c. With detergent and warm water [ ]
d. All of the above [ ]

**27. What do you think is the correct way to wash dishes in the kitchen?**

a. Soak them in water and wash them after several hours using the same water [ ]
b. Wash them immediately after the meal [ ]
c. Wash them in a basin of water and dry them with a dishcloth [ ]

**Section F: Knowledge of Food Storage and Preparation**

**28. What is the optimal temperature for storing frozen food?**

a. 4 °C [ ]
b. 0 °C [ ]
c. −18 °C or below [ ]
d. Don't know [ ]

**29. How should raw meat be preserved?**

a. Store it directly in the freezer [ ]
b. Smoke it [ ]
c. Steam it [ ]
d. Slice it into smaller pieces, seal it, and store it in the freezer [ ]

**30. Should defrosted meat be refrozen for later use?**

a. Yes [ ]
b. No [ ]
c. Maybe [ ]
d. Don't know [ ]
